# Comparing Adherence of Continuous and Automatic Positive Airway Pressure (CPAP and APAP) in Obstructive Sleep Apnea (OSA) Children

**DOI:** 10.3389/fped.2022.841705

**Published:** 2022-02-11

**Authors:** Prakarn Tovichien, Aunya Kulbun, Kanokporn Udomittipong

**Affiliations:** Division of Pulmonology, Department of Pediatrics, Faculty of Medicine Siriraj Hospital, Mahidol University, Bangkok, Thailand

**Keywords:** CPAP, APAP, CPAP adherence, CPAP tolerance, consistency of CPAP use, OSA (obstructive sleep apnoea), children

## Abstract

**Objectives:**

The treatment outcomes of pediatric obstructive sleep apnea (OSA) are affected by positive airway pressure (PAP) therapy adherence, which may be affected by the type of device used. Continuous PAP (CPAP) devices deliver a continuous and fixed air pressure level, whereas automatic PAP (APAP) devices automatically adjust the pressure to meet changing needs during sleep. The adherence, tolerance and consistency of OSA-children's use of CPAP and APAP devices were compared.

**Study design:**

One-year, observational cohort study.

**Methods:**

Twenty-seven OSA-children were enrolled. Fourteen (52%) used CPAP, and 13 (48%) used APAP. The adherence, tolerance, and consistency of the PAP usage by the two groups were compared.

**Results:**

Overall, 11 of the 27 children (41%) showed good PAP adherence. The CPAP patients averaged 4.9 h of device usage on the days used, for 60% of days, with 6 of 14 (43%) demonstrating good adherence. In comparison, the APAP patients averaged 3.2 h for 55% of days, with 5 of 13 (38%) exhibiting good adherence. The 2 groups showed no differences in their adherence, tolerance, or consistency of device usage (*P* values, 0.816, 0.609, and 0.720, respectively). Although the adherence of both groups improved in the second 6 months, it was without statistical significance (*P* values, 0.400 and 0.724). Age, sex, baseline apnea-hypopnea index, comorbidities, prescribed period, device type, mask type, and caregiver education-level were not risk factors for poor PAP adherence.

**Conclusions:**

No differences in the adherence, tolerance, or consistency of the children's use of CPAP and APAP were revealed in this small inhomogeneous cohort study with limited resources.

## Introduction

CPAP adherence effects treatment outcome of OSA-children and types of CPAP device may affect adherence based on adult study. However, our study revealed no difference of adherence, tolerance and consistency of CPAP use between OSA-children using CPAP and APAP in contrast to previous adult study.

Obstructive sleep apnea (OSA) arises from recurrent episodes of airway collapse during sleep which disrupts sleep architecture, ventilation and cardiovascular homeostasis (1). It affects 1–4% (2) of children. OSA is a common serious cause of metabolic, cardiovascular and neurocognitive morbidity in children. Most OSA-children are successfully treated with adenotonsillectomy. However, some children still have residual OSA, especially in cases with obesity, or they are simply not suitable candidates for surgery. Such patients are commonly prescribed positive airway pressure (PAP) therapy. Innovations in the PAP interfaces for children have increased the success of PAP treatment, even for young children.

In adults, PAP therapy reduces snoring, daytime sleepiness, nocturia, and subjective sleep disruption (3). Patients with a greater number of hours of PAP use tend to have a stronger feeling of being refreshed in the morning, an improved memory function, and better survival rates than otherwise. Moreover, longitudinal studies also indicate that PAP decreases the cardiovascular burden of OSA in compliant user (4). In children, PAP reduces the nocturnal and daytime symptoms of OSA. Although no randomized controlled trial on children is presently available, PAP therapy might also improve metabolic syndrome and nonalcoholic fatty liver disease (5), as well as systolic blood pressure (6).

There are two types of PAP device: continuous PAP (CPAP) and automatic PAP (APAP). CPAP delivers a continuous, fixed, air pressure throughout the breathing cycle. In contrast, the pressure delivered by APAP varies with changes in airflow resistance during sleep. The level of resistance is related to factors such as posture, degree of nasal congestion, and airway obstruction during each stage of sleep. APAP devices analyse inspiratory flow and titrate the airway pressure accordingly to maintain a constant airflow. Varying the air pressure that is required to reduce sleep disturbance may improve user comfort, thereby enhancing therapy adherence. A 2019 Cochrane review of adult studies found that people probably used APAP for 13 min longer per night at about 6 weeks compared with average usage of about 5 h per night with CPAP (4).

Tolerating PAP treatment is a highly complex issue and determined by various factors. Physical factors include disease severity, symptom relieve from PAP, underlying neurological disease and nasal anatomy. Psychological factors include locus of control, anxiety and depression (7–9). Finally, device-related factors such as mask leak, skin abrasions and nasal congestion may also deter use (10, 11).

To date, there have been few studies on PAP therapy adherence by children, and the research findings have been varied. Developmental factors may influence children's understanding of the need for therapy and its likely benefits. Moreover, high numbers of children with intellectual disabilities require PAP treatment. The efforts made by the parents of those children to initiate and sustain PAP therapy is likely to influence the level of adherence. Previous studies revealed modifiable facilitators to PAP adherence such as caregiver support, caregiver self-efficacy, authoritative parenting style, stable family structure, knowledge of PAP benefits, early adaptation to PAP and PAP apart of bedtime routine. Barriers to PAP adherence in OSA-children included poor communication between caregivers and child, discomfort of PAP interface or tubing, weight of PAP device hindering portability, lack of symptom relief/ therapeutic benefits, embarrassed about using PAP, low maternal education and older age (adolescents) (12) In addition, the minimum hours of device usage needed to achieve therapeutic benefits may also differ for children because they have a greater requirement for sleep than adults (13).

The present study set out to compare the patterns of PAP adherence (percentage of patients using device at least 4 h/day for more than 70% of nights), PAP tolerance (average hours on days used), and consistency of PAP usage (percentage of days used) of pediatric OSA patients using CPAP and APAP devices. The secondary objective was to identify the risk factors for poor PAP adherence to enable clinicians to optimize implementation and maintenance strategies for PAP therapy for children.

## Materials and Methods

This prospective cohort study included all children prescribed PAP for OSA treatment at the Department of Pediatrics, Siriraj Hospital, between 2020–2021. Our CPAP/APAP program included children with residual OSA after adenotonsillectomy and OSA related to obesity, craniofacial abnormalities or neuromuscular disorders. We diagnosed OSA from both clinical symptoms and diagnostic polysomnography (PSG) result. OSA symptoms included snoring, labored, paradoxical or obstructed breathing during sleep, sleepiness, hyperactivity, behavioral or learning problems. Diagnostic PSG revealed AHI more than 5 episodes/hour. We excluded patients with nocturnal hypoventilation (e.g. end-tidal carbon dioxide tension (PCO_2_) > 50 mmHg for > 25% of total sleep time or peak end-tidal PCO_2_ > 55 mmHg) who BiPAP was preferred (14).

After receiving printed and verbal information regarding the study, all participants provided written informed assent. In addition, the parents of the participants gave written informed consent to their children's data being included in this study.

All of the cases of PAP therapy were prescribed by a pediatric pulmonologist to treat OSA. In all, 14 CPAP devices and 13 APAP devices were used, with individual allocations being made in accordance with the patients' preferences. No humidification was used because CPAP device is not included in our national health coverage and all parents prefer to buy the cheaper one without humidifier. During the first month of usage, the devices were provided to the families free of charge and on a trial basis. If the PAP therapy was tolerated with good adherence and the family wished to continue the treatment, a payment plan was arranged. The ongoing installments were met by either the family or a supporting fund.

Typically, our PAP-therapy implementation program involved an initial, 2-night, inpatient-education session in the pediatric ward for the children and their parents. During that period, they were provided with an explanation of OSA; the principles of PAP treatment; and practical guidance on PAP desensitization, device usage, and device care. An experienced sleep technician performed the mask fitting, choosing the most appropriate mask type for each child and determining the size offering the best fit. The families were also taught how to use the ramp function of the devices. Patients with good PAP tolerance underwent a nocturnal polysomnography with CPAP titration to determine the final titration of CPAP. We used the minimal CPAP pressure able to improve the AHI to <5 episodes/h and without desaturation <90% including supine rapid eye movement (REM) sleep for therapeutic CPAP pressure. Children who accepted the placement of the mask and the trial PAP treatment then continued nocturnal home PAP at that optimum therapeutic pressure. Once home PAP-therapy had been initiated, telephone contact was made by the sleep technician once weekly to support families and troubleshoot any problems. Any side effects of the PAP therapy were identified and addressed by phone or in person as they arose. Sleep data stored in those PAP devices were downloaded each time a patient visited our clinic (every 3 months). The “hours of usage per night” were defined as the time spent at the prescribed PAP pressure. The hours were recorded for all nights of use from the first day of the PAP education session. Information from the downloaded data were used for our study. We defined “good adherence” as device usage of at least 4 h/day for more than 70% of nights.

Continuous data are presented as mean ± SD, and categorical data as frequency and percentage. The means of the groups were compared using the Mann–Whitney U test, while their proportions were compared using the chi-squared test. Data were analyzed using IBM SPSS Statistics for Windows (version 25.0; IBM Corp., Armonk, NY, USA). A *P* < 0.05 was deemed statistically significant. Before commencement of this research, its protocol was approved by the Siriraj Institutional Review Board.

## Results

During the study period, 27 patients who had been diagnosed with OSA by clinical symptoms and polysomnography were indicated for PAP treatment. Their mean age was 12.4 ± 3.2 years; they were mostly male (63%); and their mean body mass index was 27 ± 11 kg/m^2^. Of those 27 children, 11 (41%) had adenotonsillectomies; 10 (37%) had obesity; 10 (37%) had Duchenne muscular dystrophy; 10 (37%) had allergic rhinitis; 7 (26%) had Down syndrome; 5 (19%) had asthma; and 11 (41%) had intellectual disabilities that might have impacted on their ability to understand PAP treatment. In addition, 85% were snorers; 41% had a neurocognitive dysfunction; 26% had daytime fatigue; and 0% had a family history of OSA. From the baseline polysomnographies, the mean apnea-hypopnea index (AHI) was 24 ± 19 events/hour, and the mean SpO2 was 96% ± 2%. Three children (11%) had moderate OSA (defined as an AHI of at least 5 events/h), while 21 (77%) had severe OSA (defined as an AHI of at least 10 events/h). Nine children (33%) had rapid eye movement (REM)-related OSA (defined as an AHI during REM sleep of at least double that during non-REM sleep). Eleven children (41%) had positional OSA (defined as an AHI during supine sleep of at least double that during non-supine sleep). Downloaded PAP data were available for all 27 patients. The demographic data of the subjects, the PAP device brands, and the types of interface (nasal and oronasal masks) are summarized in Table 1.

**Table 1:** Demographic data.

**Age (years), mean (SD)**	**12.4 (3.2)**
**Sex**, ***n*** **(%)**	
**Male**	17 (63%)
**Female**	10 (37%)
**Comorbidities**, ***n*** **(%)**	
-Obesity	10 (37%)
-Duchenne muscular diseases	10 (37%)
-Allergic rhinitis	10 (37%)
-Down syndrome	7 (26%)
-Asthma	5 (19%)
**Post-adenotonsillectomy**	11 (41%)
**Intranasal steroids**	8 (30%)
**Type of PAP devices**, ***n*** **(%)**	
-CPAP	14 (52%)
-APAP	13 (48%)
**Brand of PAP devices**, ***n*** **(%)**	
-Philips	11 (41%)
-Resmed	7 (26%)
-Apex	6 (22%)
-Hoffrichter	2 (7%)
-Breas	1 (4%)
**Interface**, ***n*** **(%)**	
-Nasal mask	23 (85%)
-Oronasal mask	4 (15%)

Six of the 14 CPAP patients (43%) achieved good therapy adherence. The CPAP patients used their devices for an average of 4.9 h on the days used, and for 60% of days. As to the APAP patients, five of 13 (38%) showed good adherence. This group used their devices for an average of 3.2 h on the days used, and for 55% of days. The adherence (percentage of patients using device at least 4 hours/day for more than 70% of nights), tolerance (average hours on days used), and consistency of device usage (percentage of day used) by the CPAP and APAP patients did not differ (*P* values, 0.816, 0.609, and 0.720, respectively; Table 2).

**Table 2:** Adherence, tolerance, and consistency of PAP usage of CPAP and APAP groups.

	**CPAP**	**APAP**	***P* value**
	***N* = 14**	***N* = 13**	
Good adherence	6 (43%)	5 (38%)	0.816
PAP tolerance (average hours on days used)	4.9 (2.6–6.6)	3.2 (1.0–6.6)	0.609
Consistency of PAP usage (percentage of day used)	60.0% (28.4–86.0%)	55.0% (14.5–86.8%)	0.720

The CPAP group used their devices for an average of 3 h on the days used during the first 6 months of the study year. However, the average rose to 5 h during the second half of the year. A similar increase was found with the APAP group. Usage climbed slightly from an average of 2.2 h during the first half of the study year to 2.5 h during the second half. Nevertheless, the trends shown by the CPAP and APAP groups toward increasing PAP adherence in the second half of the study year were without statistical significance (*P* values, 0.400 and 0.724, respectively).

Overall, 11 children exhibited good PAP-therapy adherence, whereas 16 showed poor adherence. Apart from discomfort arising from the use of a PAP device, all other characteristics of the patients in the two groups were similar (Table 3). The only risk factor for poor PAP-therapy adherence identified by this study was the discomfort associated with PAP device usage (Table 4). We interviewed the 16 children who had demonstrated poor therapy adherence to ascertain the underlying reasons. None reported that their poor adherence was related to complications such as congestion or skin problems.

**Table 3:** Characteristics of users with good vs poor PAP-therapy adherence.

	**Good adherence**	**Poor adherence**	***P* value**
	***N* = 11**	***N* = 16**	
Age (years)	12.54 (3.17)	12.31 (3.24)	0.855
Male, *n* (%)	6 (54%)	11 (69%)	0.687
Comorbidities, *n* (%)			
-Obesity	2 (18%)	8 (50%)	0.124
-Duchenne muscular diseases	5 (45%)	5 (31%)	0.687
-Allergic rhinitis	3 (27%)	7 (44%)	0.448
-Down syndrome	4 (36%)	3 (19%)	0.391
-Asthma	2 (18%)	3 (19%)	1.000
-Intellectual disability	6 (55%)	5 (31%)	0.264
Previous adenotonsillectomy, *n* (%)	5 (46%)	6 (38%)	0.710
Intranasal steroids use, *n* (%)	3 (27%)	5 (31%)	1.000
OAHI prior to treatment	20.5 (14.5–26.5)	17.5 (9.5–25.5)	0.651
Type of PAP devices, *n* (%)			
-CPAP	6 (54%)	8 (50%)	1.000
-APAP	5 (46%)	8 (50%)	
Brand of PAP devices, *n* (%)			
-Phillip	3 (27%)	8 (50%)	0.163
-Resmed	2 (18%)	5 (31%)	
-Apex	5 (46%)	1 (6%)	
-Hoffrichter	1 (9%)	1 (6%)	
-Breas	0 (0%)	1 (6%)	
Interface, *n* (%)			
-Nasal mask	9 (82%)	14 (88%)	1.000
-Oronasal mask	2 (8%)	2 (12%)	
Duration of PAP usage, media*n* (IQR)	36.5 (19.3–46.6)	22.8 (10.4–47.9)	0.916
Parental involvement, *n* (%)	11 (100%)	13 (81%)	0.128
Parental education, *n* (%)			
-Primary school	1 (9%)	4 (25%)	0.632
-High school	2 (18%)	4 (25%)	
-Higher than bachelor's degree	8 (73%)	8 (50%)	
Discomfort from PAP, *n* (%)	0 (0%)	11 (69%)	<0.005^*^
Lack of clinical benefits, *n* (%)	0 (0%)	5 (31%)	0.060

* *A p-value < 0.05 indicates statistical significance*.

**Table 4:** Risk factors for poor PAP-therapy adherence.

	**OR (95% CI)**	***P* value**
Age	0.98 (0.76–1.30)	0.850
Male sex	0.54 (0.11–2.67)	0.455
Baseline AHI	0.99 (0.95–1.03)	0.650
Oronasal mask	0.64 (0.76–5.42)	0.685
Lower maternal education	0.38 (0.07–2.00)	0.244
Discomfort from PAP	48 (2.37–973.97)	0.012^*^
Lack of clinical benefits	3.33 (0.32–34.83)	0.315

* *A p-value < 0.05 indicates statistical significance*.

## Discussion

In adults, PAP usage for >4 h per night is associated with improvements in apnea-hypopnea indices and Epworth Sleepiness Scale scores. There is also a linear relationship between the hours of nightly PAP-device usage and improvements in OSA symptoms, with a leveling off at approximately 7 h of use and no further gains in benefits thereafter (15, 16).

In the case of children, PAP adherence rates are typically quite poor, with most studies having reported usage averages of between 3 and 4 h per night (12). However, Ramirez and associates observed high levels of PAP usage (>8 h per night). In their study, PAP therapy was implemented in a dedicated, pediatric, noninvasive ventilation unit, and the patients and caregivers were provided with clinical and behavioral support (17). Another study suggested that it may not be reasonable to apply the definition of adherence used for adults to children. This is because children have longer recommended and actual sleep durations than adults, and those durations vary with age (13). Drawing on this hypothesis, we designed our PAP education program to include family support. We then conducted this research to determine whether PAP-therapy adherence differed between patients using CPAP and APAP devices.

Our study supported the findings of previous studies that there is generally poor PAP-therapy adherence in overall children. Those investigations reported that between only 41 and 75% of children showed good adherence (defined as an average of at least 4 h on 70% of the nights) (18–21). Because our hospital has a center of excellence for neuromuscular diseases, many of our cases needed caregiver assistance in putting on the PAP interface. The efforts made by the caregiver to sustain PAP therapy is likely to influence the level of adherence.

The present work found no differences in the adherence, tolerance, or consistency of the PAP therapies of the CPAP and APAP groups. This contrasts with a meta-analysis of adult studies, which identified a statistically significant difference of 11 min per night favoring APAP (22).

Our results showed that the major barrier to good adherence was not associated with the baseline characteristics of the patients, PAP device, or complications of device usage. Instead, the major barrier was related to the discomfort associated with PAP device usage. Personalized desensitization programs and behavioral interventions were shown by a previous investigation to increase the hours of usage in a group of children who had poor PAP-therapy adherence (23–25). In the present work, the adherence of both groups tended to improve in the second half of the study year, albeit without statistical significance. Ways that could be utilized to improve PAP-therapy adherence include education about the benefits of PAP usage and desensitization techniques prior to therapy initiation, play therapy, cognitive behavioral therapy for older children, positive reinforcement, and parental support using tele-education and telemonitoring.

The clinical benefits of PAP therapy in adults are directly related to the hours of usage of the devices per night. Skipping a night of treatment leads to a return of daytime sleepiness. In the case of children, untreated OSA is known to have adverse neurocognitive and behavioral consequences. Furthermore, improvements in PAP adherence have been associated with improvements in the parent-reported symptoms in children receiving PAP therapy. The impact of PAP therapy on the symptoms that can be perceived by the children themselves may play a key role in adherence.

A previous study demonstrated a trend toward an association between high PAP adherence (in terms of hours of use per night) and a younger age, a high AHI at diagnosis, primary vs middle/high school attendance, and neurocognitive disorders at baseline (26). However, the predictors of poor adherence are probably specific to each population. Unfortunately, data are scarce on the long-term PAP-therapy adherence of children and the factors influencing that adherence.

As to our secondary objective, we did not find any predictors for poor CPAP adherence. More specifically, the following factors showed no association: age, sex, baseline AHI, comorbidities, a previous adenotonsillectomy, PAP-device type, interface type, and education level of the caregiver.

We observed no association between age and PAP-therapy adherence. However, this finding contrasts with the work of DiFeo and colleagues (27). Likewise, another study suggested that educational programs for pediatric patients and their families should differ with age to improve PAP adherence (25). Other research also concluded that it was particularly important that adolescents with OSA be well supported in their use of PAP therapy (28).

Some studies revealed that female sex, developmental delay (18), and maternal education (27) were associated with a good adherence. Like some other studies, no such associations were demonstrated in our study population (29).

We also observed no association between OSA severity and PAP adherence. This corresponded with the results of the retrospective study by Hawkin et al. (18). It reported that OSA (diagnostic AHI and degree of hypoxemia), therapeutic pressure, and residual AHI had no impact on PAP adherence.

Specific integrated care support at home serves as an important way to improve self-efficacy when starting PAP therapy in children with OSA (30).

We acknowledge that our study had a small inhomogeneous sample and might therefore lacked power to achieve statistical significance. We performed this study in a place with limited resources. Our national health coverage doesn't include cost of CPAP device. Parents had to buy CPAP on their own and most parents preferred to buy the cheaper one without humidifier. Although bi-level PAP device would be more efficient for children with Duchenne muscular dystrophy (DMD), we didn't have enough financial support for them. The diagnostic PSG of all children with DMD in this study had only moderate OSA without sleep-related hypoventilation. We repeated PSG of them annually and switched to BiPAP in case that we found progressive severity of OSA or evidence of sleep-related hypoventilation. The results must be considered in the light of this important limits.

## Conclusions

No differences in the adherence, tolerance, or consistency of the PAP therapies of the CPAP and APAP groups were revealed in this small inhomogeneous cohort study with limited resources.

## Data Availability Statement

The original contributions presented in the study are included in the article/supplementary material, further inquiries can be directed to the corresponding author.

## Ethics Statement

The studies involving human participants were reviewed and approved by Institutional Review Board of Siriraj Hospital. Written informed consent to participate in this study was provided by the participants' legal guardian/next of kin.

## Author Contributions

PT contributed to study conception, study design, statistical analysis, and manuscript preparation. AK recruited study participants, conducted fieldwork, and performed data collection. KU contributed to study conception and design. All authors contributed to the article and approved the submitted version.

## Conflict of Interest

The authors declare that the research was conducted in the absence of any commercial or financial relationships that could be construed as a potential conflict of interest.

## Publisher's Note

All claims expressed in this article are solely those of the authors and do not necessarily represent those of their affiliated organizations, or those of the publisher, the editors and the reviewers. Any product that may be evaluated in this article, or claim that may be made by its manufacturer, is not guaranteed or endorsed by the publisher.
